# Tension-induced twist of twist-spun carbon nanotube yarns and its effect on their torsional behavior

**DOI:** 10.1038/s41598-018-24458-0

**Published:** 2018-04-18

**Authors:** Seung-Yeol Jeon, Dongil Kwon, Woong-Ryeol Yu

**Affiliations:** 1 Department of Materials Science and Engineering and Research Institute of Advanced Materials (RIAM), Seoul National University, Seoul, Korea; 2 Present Address: Center for Computational Science, Korea Institute of Science and Technology (KIST), Seoul, Korea

## Abstract

Twist-spun carbon nanotube (CNT) yarns exhibit a large and reversible rotational behavior under specific boundary conditions. *In situ* polarized Raman spectroscopy revealed that a tension-induced twist provides reversibility to this rotation. The orientation changes of individual CNTs were followed when twist-spun CNT yarns were untwisted and subsequently retwisted. Twist-spun CNT yarn, when untwisted and subsequently retwisted under the one-ended tethered boundary condition, showed irreversible orientation changes of the individual CNTs due to snarls formed during the untwisting operation, which resulted in macroscopic irreversible rotational behavior of the CNT yarns. In contrast, the orientation changes of the individual CNTs in twist-spun CNT yarn, when operated under the two-ended tethered boundary condition, were hysteretically reversible due to a tension-induced twist, which has not been reported previously. Indeed, the tension-induced twist was observed by following the orientation change of individual CNTs in elongated CNT yarns, which simulated the deformational behavior of the CNT yarn rotated under the two-ended tethered boundary condition.

## Introduction

Twist-spun carbon nanotube (CNT) yarns have been suggested as an efficient route to transport the outstanding electrical, thermal, and mechanical properties of CNTs at the nanoscale to the micro/macro scales^1^. CNT yarns are strong and tough, flexible and electrically conductive, and are used in various applications including mechanical reinforcements for polymer composites^2^, flexible electronic devices (e.g., flexible supercapacitors, electrodes and fiber solar cells)^3–5^, and flexible actuator and sensors^6,7^. The torsional behavior of CNT yarns has been widely studied due to their large rotational behavior when actuated. The helix angle of CNT yarns, i.e., the CNT orientation on the yarn surface, is a key parameter to determine their actuation properties^7,8^. CNT yarns have been structurally modified into coiled yarns^9^, dual-Archimedean yarns, and their combinations as S and Z yarns^10,11^ to maximize the actuation properties. Moreover, CNT yarns have been hybridized with various polymers, such as paraffin wax^8–10^, copolymers^8,11^, and solid gels^12,13^, resulting in improved actuation properties due to extensive swelling of guest materials.

Interestingly, the orientation angle of CNTs (i.e., angle between CNTs and the CNT yarn axis) of twist-spun CNT yarns increases along with the large radial expansion of yarns during untwisting, but the angle returns to its original configuration upon retwisting^7,10^; this cannot be explained by classical yarn mechanics^14^. Common twist-spun yarns such as wool and cotton yarns show decreased fiber orientation angle and radial contraction when untwisted. A previous study explained that electrolyte injection (i.e., electrochemical charge injection) and applying a voltage produce this unusual phenomenon^7^. Since then, however, it has been reported that the same phenomenon occurs against photonic or electrical external stimuli without any electrolyte or other liquid infiltration^10,15^, suggesting that the torsional behavior of CNT yarns is attributed to the inherent mechanical properties of CNT yarns themselves. Unfortunately, the torsional mechanism of CNT yarns has been differently described, depending on the system which researchers employed^7,10,15^. Therefore, a mechanism should be revealed that can explain the torsional behavior of CNT yarns regardless of systems. In order to clarify the torsional behavior of the CNT yarn, it is important to analyze the movement of the internal CNTs, e.g., the change in the orientation angle, during torsion. Few analytical studies have been carried out to investigate the orientation change of individual CNTs within a CNT yarn or its effect on the actuation properties, even though the orientation of CNTs is the most important factor in determining the actuation behavior of CNT yarns^16^. Even in even relevant papers, the actuation mechanism of a CNT yarn, including large and reversible rotation, has not been explained well because only the change in the orientation angle of CNTs on the surface of the yarns was examined^11,17,18^.

Polarized Raman spectroscopy has been used to characterize the orientation of CNTs because the intensity of the G band depends on the orientation of the CNT, i.e., as the orientation angle of the nanotube axis increases with respect to the polarizer direction, the intensity decreases monotonously^19,20^. The orientation angles of CNTs in the CNT yarns were calculated using two intensities, which were measured by aligning the polarizer parallel and perpendicular to the CNT yarn axis^21^. The calculated results were represented by the orientation distribution function (ODF) indicating the probability of an individual CNT locating at the specific orientation. The effect of the twist angle of CNT yarns and the incidence angle of the Raman laser on the intensity change of the Raman spectrum were investigated^16^. The deformation of individual CNTs within CNT yarns was also characterized by investigating the peak shifts for both unstrained and strained CNT yarns^22,23^. The axial strain of the CNTs within CNT yarns was evaluated quantitatively through a strain transfer factor^24^.

**Figure 1:**
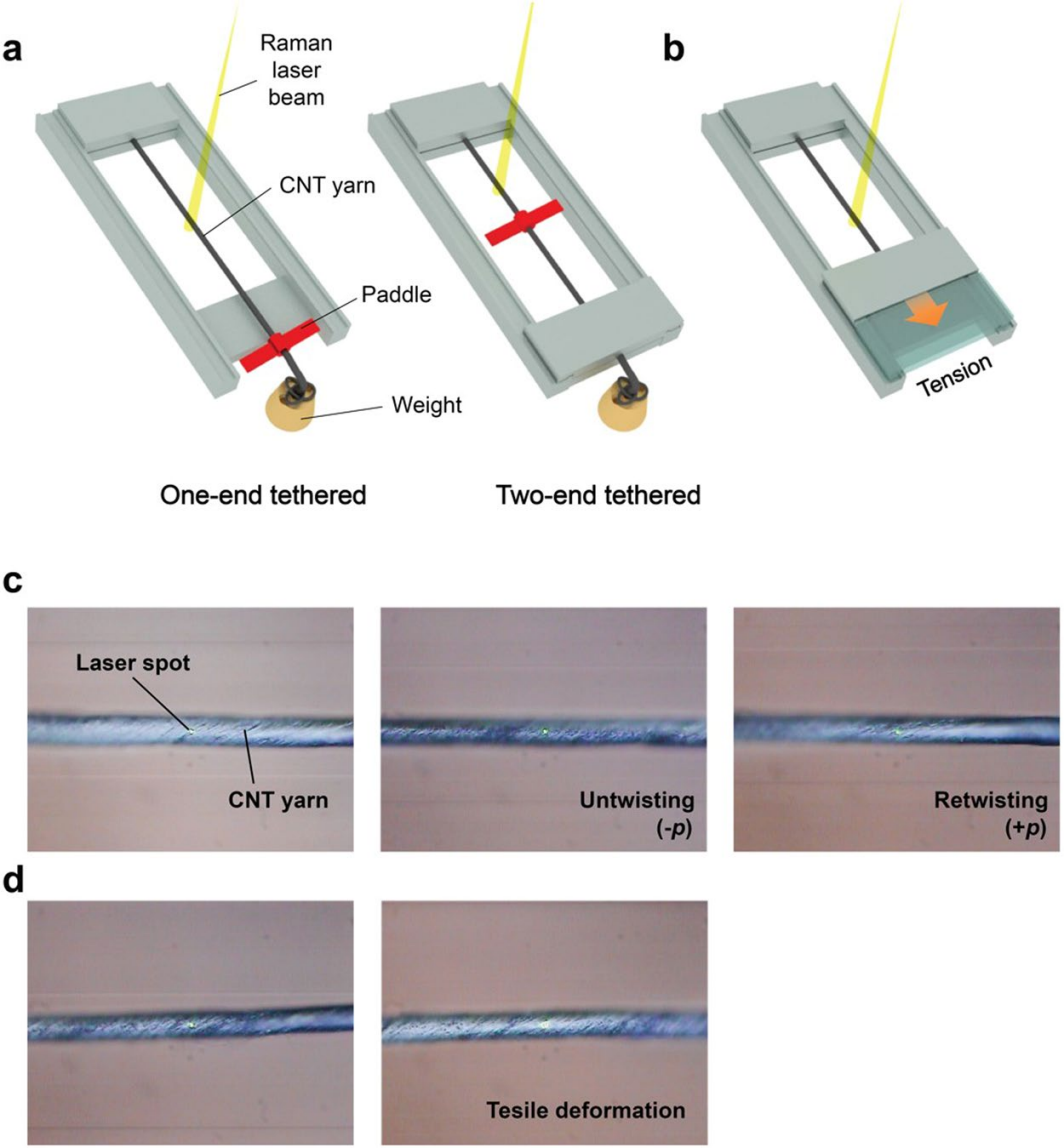
*In-situ* Raman spectroscopy system to characterize the orientation and deformation of individual CNTs in CNT yarns. (**a**,**b**) Schematic diagrams of (**a**) torsional and (**b**) tensile devices used for *in-situ* Raman spectroscopy studies for characterizing the orientation and deformation of individual carbon nanotubes (CNTs) in CNT yarns. (**c**,**d**) *in-situ* Raman tests during (**c**) torsional and (**d**) tensile loading.

Few studies were carried out to quantitatively characterize the average orientation change of individual CNTs while CNT yarns are actuated. In this study, the actuation behavior of CNT yarns, which were manufactured by twisting CNT sheets drawn from CNT forest, was investigated using *in-situ* Raman spectroscopy. Raman spectra were obtained from CNT yarns when they were twisted or tensioned by external forces (Fig. 1). Then, the average orientation of individual CNTs were quantitatively determined, from which the actuation mechanism of twist-spun CNT yarns was revealed.

## Results

### Torsional behavior of CNT yarns

**Figure 2:**
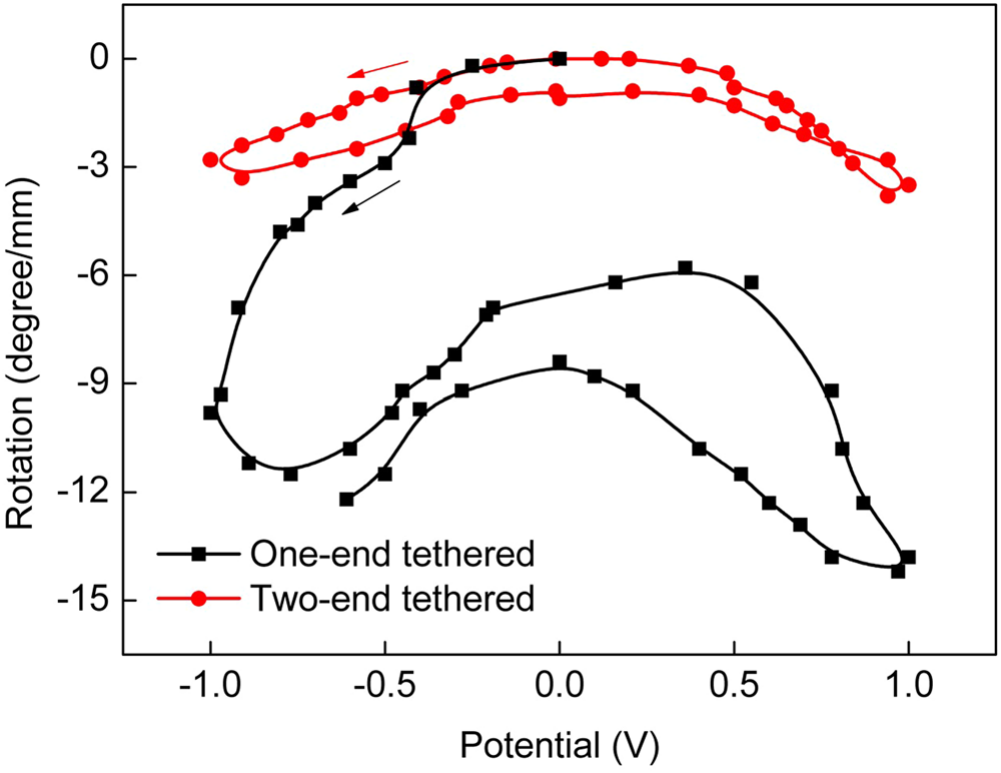
Schematic plot of the torsional behavior of the CNT yarn when immersed in the electrolyte and actuated by the electric potential, clearly demonstrating the reversible rotation in the two-ended tethered CNT yarns. The black line represents the irreversible rotation of the one-ended tethered CNT yarns. This figure was redrawn from Figure S2 in the literature^7^.

The torsional behavior of CNT yarns when actuated strongly depends upon the boundary conditions. Figure 2 (which is schematically drawn from ref.^7^) shows that one-ended tethered CNT yarns rotate much more than two-ended tethered yarns. However, reversible rotation is only observed in two-ended tethered CNT yarns. What initiates such reversible or irreversible rotational behavior of CNT yarn? We investigated this obtaining *in situ* Raman spectra during torsional testing of CNT yarns (see Methods section for detailed experiments) as follows.

**Figure 3:**
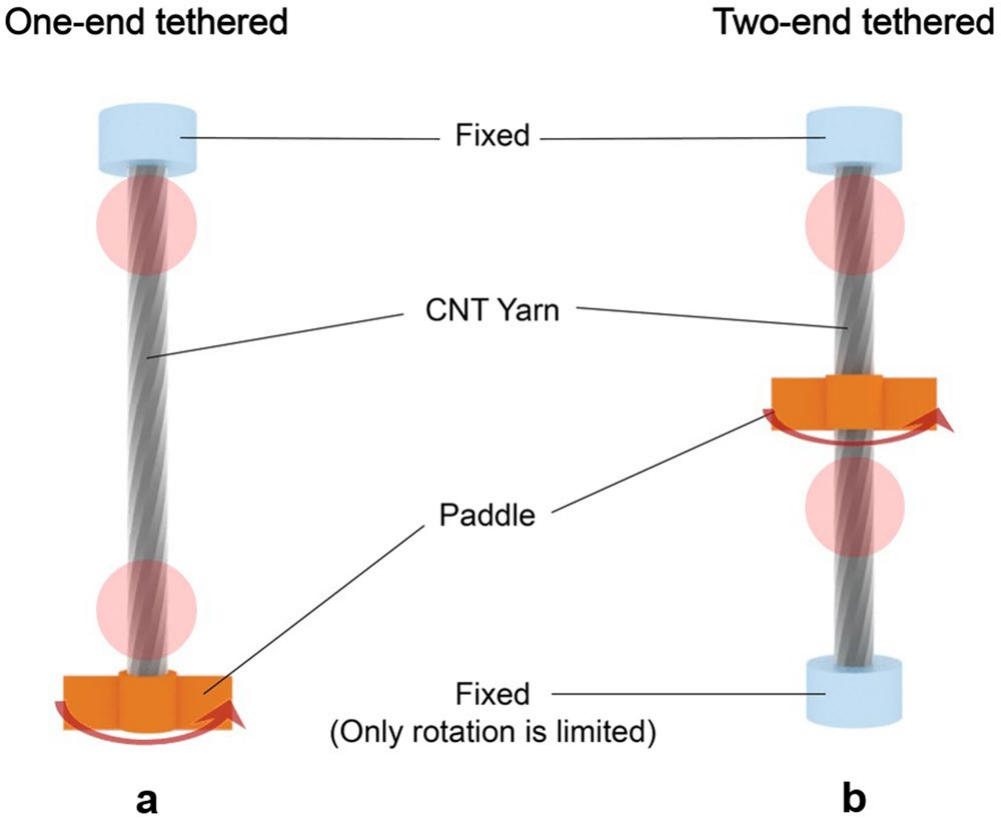
*In situ* torsional testing of CNT yarns. (**a**) One-ended tethered configuration, i.e., one end of the CNT yarn is fixed while twisting/untwisting operations are applied at the other end via a paddle. This configuration allows the CNT yarn to move along the yarn axis. (**b**) Two-ended tethered configuration, i.e., one end of the CNT yarn is fixed as in the one-ended tethered configuration while the other end is fixed to prevent rotation but allow movement along the yarn axis. In this configuration, twisting/untwisting operations are applied at the center part of the CNT yarn via a paddle. The red solid circles represent the area where the orientation changes of individual CNTs were measured using Raman spectroscopy.

**Figure 4:**
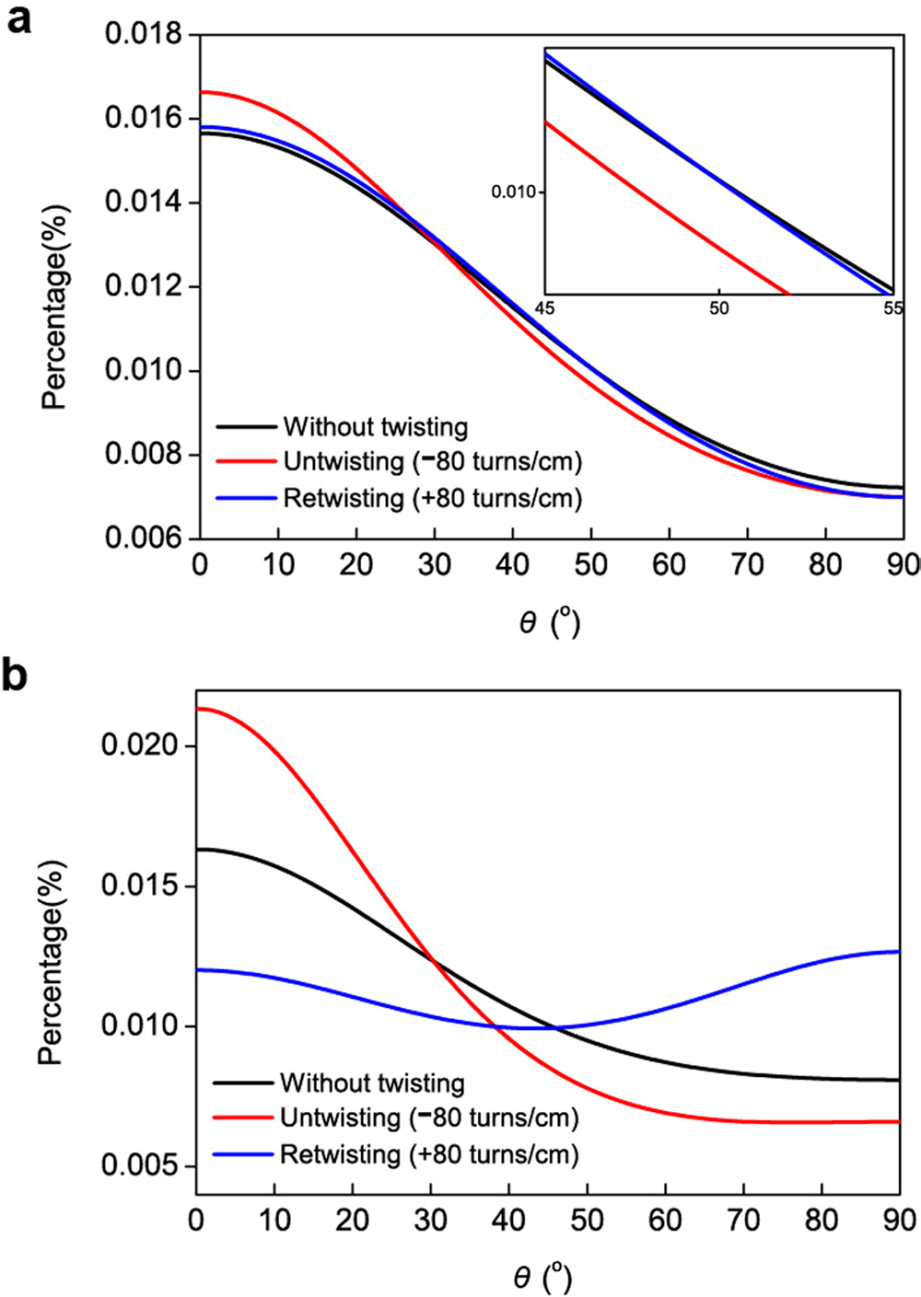
Orientation angles of individual CNTs in the one-tethered CNT yarn. (**a**) CNT orientations obtained around the fixed end of the CNT yarn without twisting or untwisting, with untwisting (−80 turns cm^−1^) and with twisting (+80 turns cm^−1^). (**b**) CNT orientations obtained near the free end without twisting or untwisting, with untwisting (−80 turns cm^−1^) and with twisting (+80 turns cm^−1^).

Given that the rotation of one-ended tethered yarns depends on the distance from the fixed region^7^, the orientation change of individual CNTs was observed at two points of the CNT yarn, i.e., near the fixed end and the free end where untwisting and twisting operations were applied (Fig. 3). Figure 4a shows decreased orientation angles of individual CNTs around the fixed end of CNT yarns when untwisted, which is typical behavior of twist-spun yarns. Note that the reduced orientation angle represents more CNTs aligned along the CNT yarn axis. Because the CNT yarns had been manufactured by twisting the CNT sheet drawn from CNT forests, they were already in a twisted state before the untwisting operation was applied. The untwisting operation returned the CNTs in the yarn back to the untwisted state, and the orientation angle decreased. Upon subsequent twisting operation, the orientation angle increased again (Fig. 4). Near the free end of CNTyarns, however, unexpected orientation angle changes were observed (Fig. 4b). Upon untwisting, a large number of CNTs changed their orientation angles and, thus, their distribution. The CNTs did not return to their original state upon retwisting. This behavior is totally different from the orientation changes of individual CNTs occurring near the fixed end. Note that the rotation depends on the distance from the fixed region through an equation, $\varphi (x)=x{\rm{\Delta }}\theta$ ^7^, where *ϕ* is the rotation, *x* is the distance from the tethered end to the rotated part, and Δ*θ* is the rotation per yarn length. Therefore, more rotation is indeed applied to the free end part where rotation is applied, so that the orientation angle change of this part is larger. Therefore, the magnitude of the change in the orientation angle itself does not account for different torsional behavior because rotation amount depends on position and the boundary conditions. The morphological changes of the CNT yarn observed by scanning electron microscopy (SEM) suggested that, upon untwisting, some part of the CNT yarns near the fixed end experienced a kind of buckling, as shown in Supplementary Figure 2b. Typically, twisted yarns rotate with the yarn axis of rotation under external torsional loading. When the yarn is untwisted, strong elastic forces developed during the twisting operation are released. Thus, the yarn axis is coiled or moves away from the axis of rotation, producing yarn snarls^25^ (Supplementary Figure 2d). As such, when the CNT yarn was untwisted, the high torque that developed during the twisting operation was released, forming yarn snarls. When retwisting was applied, these yarn snarls did not return to the original yarn state (Supplementary Figure 2c), thus bringing about the irreversible CNT orientations (Fig. 4f) and finally the irreversible rotational behavior, as shown in Fig. 2.

**Figure 5:**
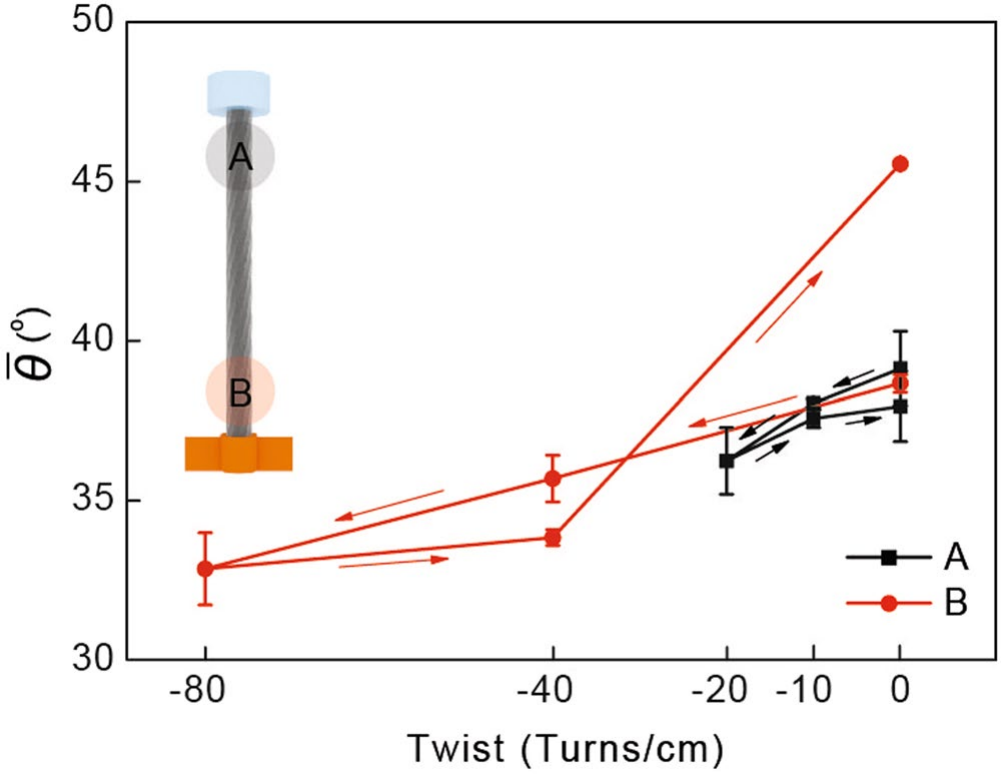
Change in average orientation angle of one-ended tethered yarn during torsional test. Arrows indicate untwisting and retwisting operations. Legends A and B indicate the measurement regions as shown in the inset figure.

The change in the average orientation angle, which was dependent on the twisting operation, was calculated for one-ended yarns (Fig. 5). The average orientation angle was calculated by averaging the multiplication of the orientation angles and their percentages from the data in Fig. 4. Figure 5 shows that the change of the orientation angle was larger near the paddle than at the fixed region, which is consistent with basic torsion theory, i.e., the torsion angle increases linearly from the fixed end to the other end where a twisting operation is applied. Actual angle changes were 0.094° (standard deviation: 0.028°) per twist cm^−1^ for the one-ended tethered yarns. The average orientation angle of the one-ended tethered CNT yarn changed linearly, in particular, near the paddle, when untwisted. This linearity was not maintained upon subsequent twisting due to the snarl formation, as shown in Fig. 5.

**Figure 6:**
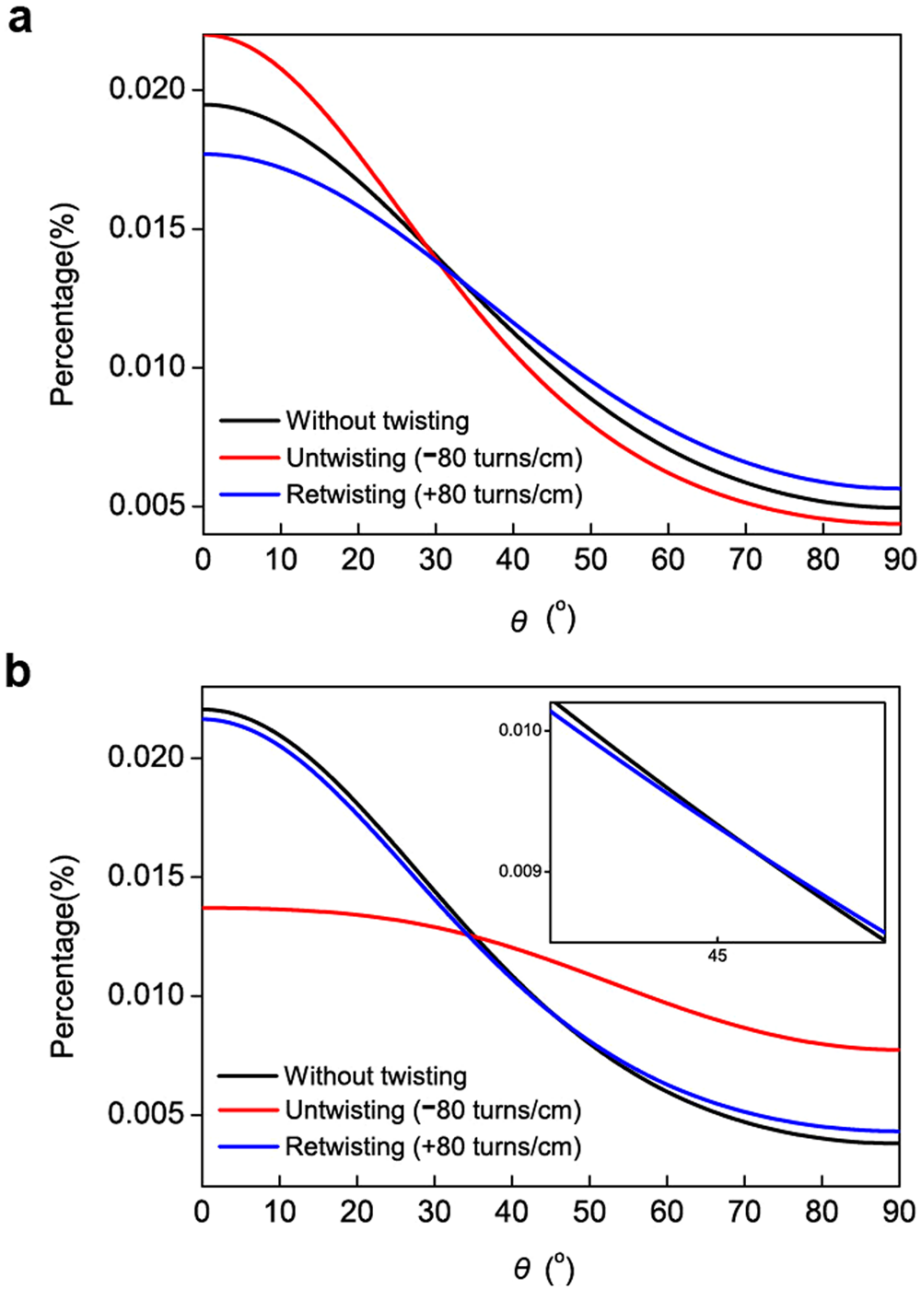
Orientation angle of individual CNTs in two-tethered CNT yarn (Fig. 3(b)). (**a**) CNT orientations obtained around the fixed end of the CNT yarn without twisting or untwisting, with untwisting (−80 turns cm^−1^) and with retwisting (+80 turns cm^−1^), respectively. (**b**) CNT orientations obtained around the middle between the lower fixed end and the yarn center.

**Figure 7:**
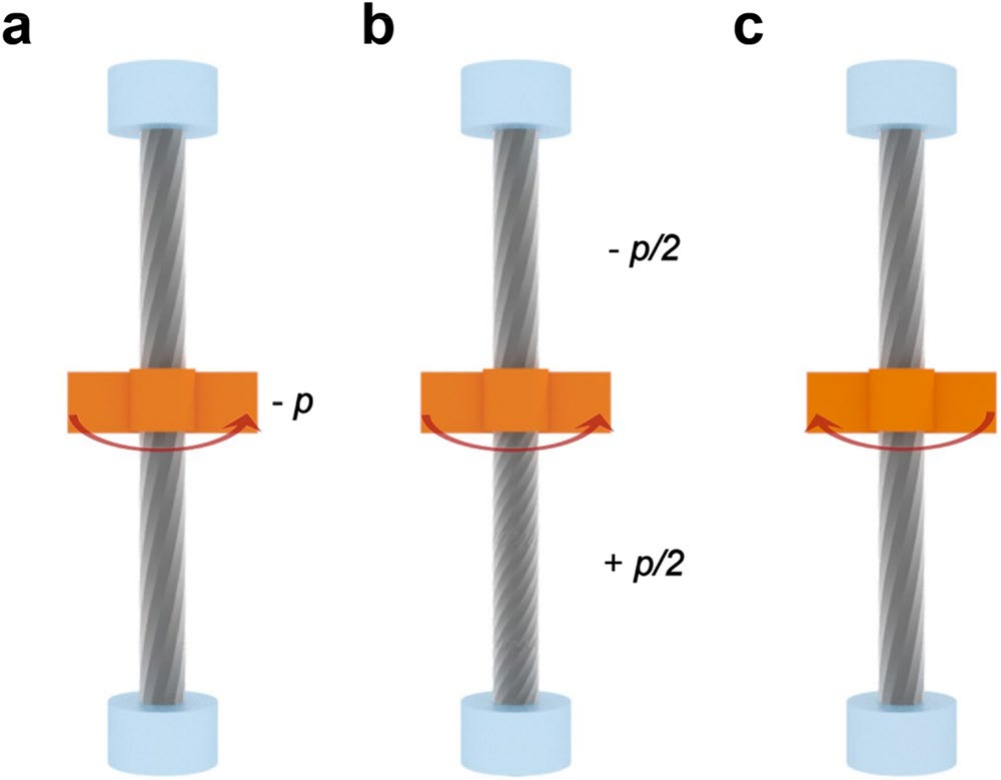
Schematic diagram explaining the torsional behavior of the two-ended tethered CNT yarn. (**a**) The untwisting operation is applied, (**b**) resulting in untwisting in the upper part and further twisting in the lower part. (**c**) Subsequent twisting returns the yarn to the original configuration.

**Figure 8:**
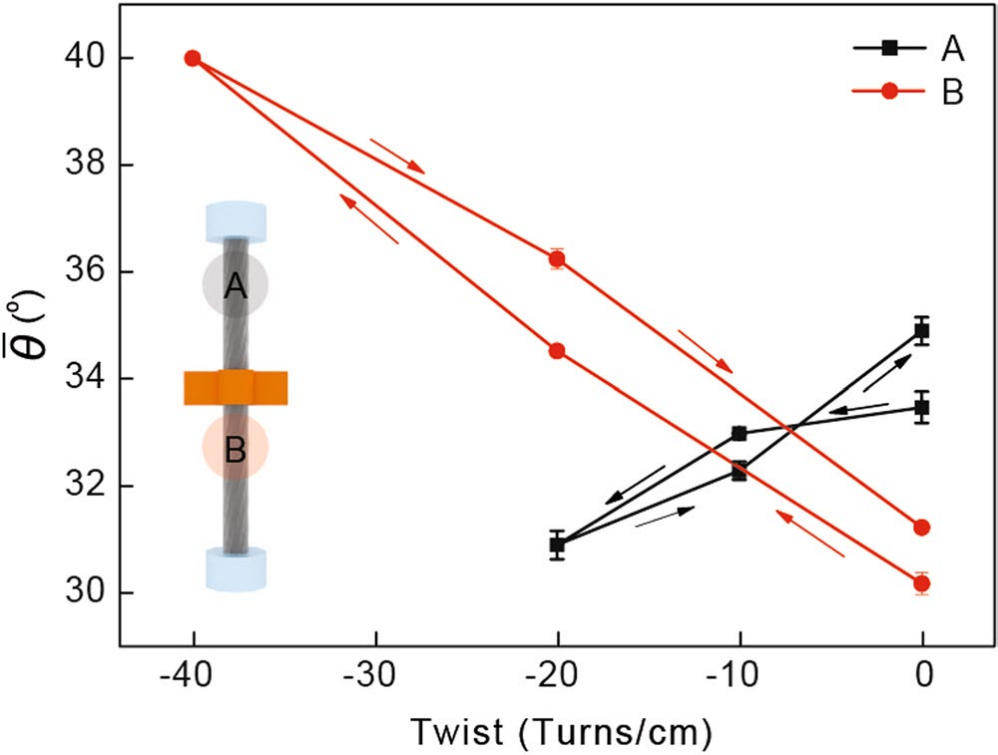
Change in the average orientation angle of the two-ended tethered yarn during untwisting and retwisting operations. Arrows indicate the untwisting and retwisting directions. Legends A and B indicate the measurement regions as shown in the inset.

The orientation angle changes of individual CNTs were also observed for the two-ended tethered CNT yarn when untwisting and twisting operations were applied at the center of the CNT yarn (Fig. 3b). The orientation angle changes of individual CNTs were measured near the fixed end and the lower middle portion of the CNT yarn (Fig. 6). Typical behavior was observed near the fixed end, i.e., the orientation angle of individual CNTs decreased upon untwisting and increased upon subsequent retwisting (Fig. 6a). As mentioned in the one-tethered yarn case, this is typical behavior of twist-spun yarns. An interesting phenomenon was observed around the lower middle portion of the CNT yarn. Upon untwisting, the orientation angle of the individual CNTs increased and then decreased upon subsequent retwisting (Fig. 6b), which is opposite to the phenomenon that we observed around the fixed end of the CNT yarn. This implies that the rotational direction of individual CNTs near the fixed end and the lower middle portion of the CNT yarn was opposite when untwisting and subsequent retwisting operations were applied at its center. Figure 7a,b explain this behavior upon untwisting as follows: the upper part of the two-tethered CNT yarn was untwisted (i.e., the orientation angle decreased) while its lower part was further twisted (i.e., the orientation angle increased). Figure 7c indicates that subsequent retwisting can return the two-tethered yarn to the original twisted state. This reasoning was investigated further using the averaged orientation angle of the two-ended tethered yarn calculated from Fig. 6. Figure 8 shows that the change in the orientation angle was larger near the paddle than the fixed region, as in the one-tethered yarn case. The actual angle change was 0.25° (standard deviation: 0.0092°) per twist cm^−1^, which is much larger than the angle change in the one-tethered yarn in Fig. 5. The change of average orientation angle in Fig. 8 has three interesting aspects. First, the orientation change of individual CNTs depicted in Fig. 7a,b was clearly observed. Second, hysteretic behavior during untwisting and retwisting operations was clearly observed as reported in the literature (see Fig. 2 for a schematic explanation), even though hysteretic macroscopic rotation of CNT yarn was observed (see the left part of the actuation curve of the two-ended tethered CNT yarns in Fig. 2). Third, additional twist was introduced during the untwisting and twisting operations: the end points are higher than the starting points in Fig. 8. This additional twist was not reported and discussed elsewhere, even though it can be indirectly observed in Fig. 2, i.e., compare the *y*-coordinate at zero potential after the untwisting and retwisting operations. Finally, it is claimed that Fig. 7c does not occur in twist-spun CNT yarn. It is interesting that the macroscopic rotational behavior of the two-tethered CNT yarn was reversible during an actuation consisting of untwisting, retwisting, further retwisting (second twisting), and a second untwisting, even though hysteretic rotational behavior was observed during the first untwisting and retwisting operations. We suggest a mechanism that can explain such a reversible rotational behavior of the two-tethered CNT yarn as follows.

**Figure 9:**
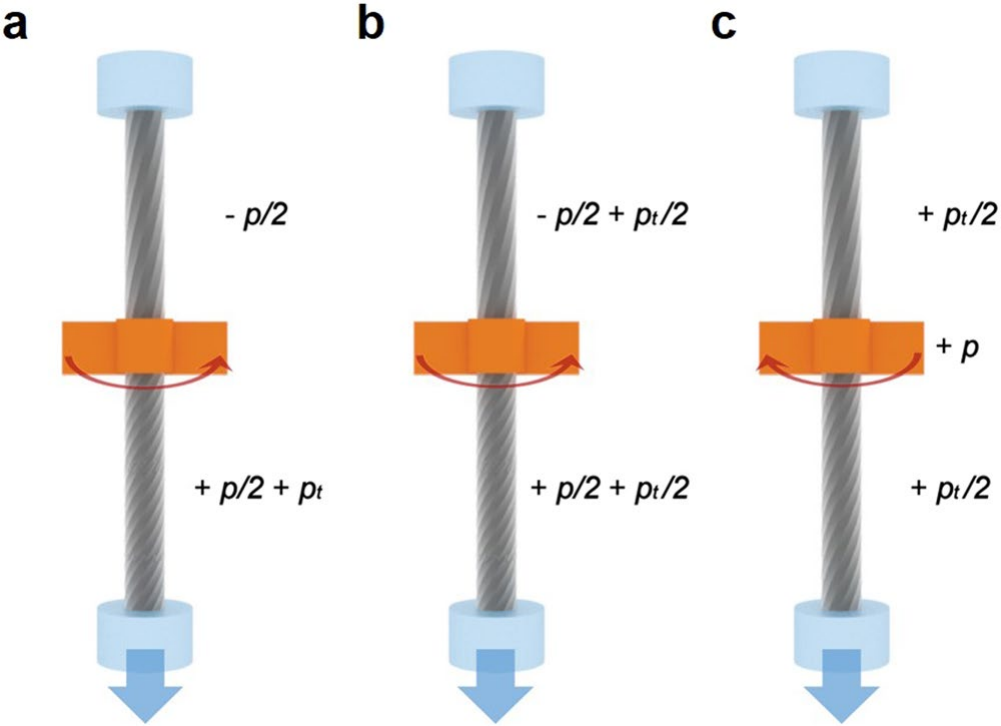
Schematic diagram explaining the rotational behavior of the two-ended tethered CNT yarn. (**a**) The applied untwisting operation under tension leads to additional twisting (*p*_*t*_). (**b**) Half of the additional twist is transferred to the upper part for torsional equilibrium. (**c**) Subsequent retwisting removes the additional twist after the untwisting and retwisting operations. This additional twist is called tension-induced twist.

When an untwisting operation is applied to the CNT yarn at its center, during which tension is applied to the lower part of the CNT yarn, an additional twist is introduced to the lower part of the yarn due to the torque developed from the tension (Fig. 9a). This is consistent with the observations in Fig. 6a,b, i.e., asymmetric and opposite rotation of CNTs. For torsional equilibrium, half of the additional twist is then transferred to the upper part (Fig. 9b). When the CNT yarn is retwisted by the same amount of untwisting angle, a twist remains at both parts of the CNT yarn (Fig. 9c). Note that the detailed twist configuration (and thus CNT orientation) in Fig. 9c is different from that in Fig. 9a, even though a macroscopic rotation is cancelled by applying the same amount of untwist and retwist. The tension-induced twist can explain the hysteretic rotation of the two-ended tethered yarn in Fig. 2. Further retwisting and untwisting operations, which are the last two steps of the actuation (i.e., the right curve of the actuation curve in Fig. 2), are next considered, focusing on tension-induced twist.

**Figure 10:**
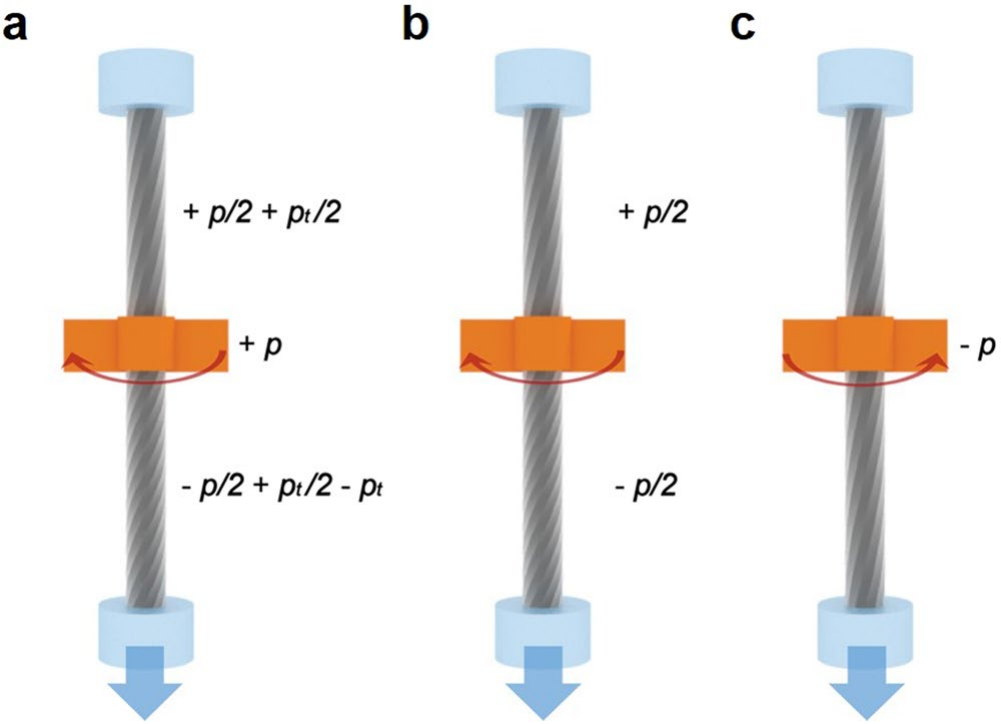
Schematic diagram explaining the rotational behavior of the two-ended tethered CNT yarn. (**a**) Additional untwist (−*p*_*t*_, tension-induced untwist) is introduced to the lower part of the CNT yarn when further retwisting (+*p*) proceeds. (**b**) Additional untwist is transferred from the upper part to the lower part, cancelling the additional twist developed in the previous untwist in Figs 8c and 9b. The CNT yarn returns to the original state as the second untwisting operation proceeds.

With further twisting operation (+*p*), the upper part of the CNT yarn is twisted (+*p*/2) while its lower part is untwisted (−*p*/2) (Fig. 10a), and is accompanied by a tension-induced untwist (−*p*_*t*_). Note that the tension-induced twist (*p*_*t*_/2) remained as a result of the untwisting operation under tension in Fig. 9c. Half of the additional twist in the lower part is transferred to the upper part for torsional equilibrium, cancelling the additional twist developed in the previous untwisting operation in Fig. 9b (Fig. 10b). Finally, the CNT yarn returns to its original configuration upon further untwisting (second untwisting) (Fig. 10c). Again, note that the twist configuration in Fig. 10c is different from that in Fig. 10a, even though macroscopic rotation is cancelled by applying the same amount of twist and untwist. This different CNT orientation brought about the hysteretic behavior of the right-hand curve in Fig. 2. Notably, the twist configuration (and CNT orientation) in Fig. 10c is the same as that of Fig. 7a. In fact, due to this mechanism, the two-tethered yarn can rotate reversibly and repeatedly, as reported elsewhere^7,15^. Tension-induced twist is further discussed in the following section.

### Tensile behavior of CNT yarns

Tension-induced twist was observed directly by obtaining *in situ* Raman spectra during the tensile testing of the CNT yarns (see Methods section). To further investigate their tensile behavior, in particular the internal changes of the yarn such as the orientation and deformation of individual CNTs, CNT yarns with a diameter of about 10 μm were tensioned and analyzed using *in situ* polarized Raman spectroscopy. Considering the penetration depth (465 nm) of the Raman laser (see Methods section), CNT yarns with a diameter of less than 1 μm were also investigated under applied tension; it was anticipated that such analysis would include the internal changes of the CNT yarn, including its core part.

Supplementary Figure 3 shows the distribution of the orientation angles of the individual CNTs in the CNT yarn near its surface. At low strain (e.g., 0.9%), the orientation angle of the CNTs increased slightly, i.e., the CNTs tended to rotate away from the tensile axis, proving that tension-induced twist happened in the CNT yarn. Note again that zero orientation angle indicates that the CNT is perfectly aligned with the yarn axis (or the tensile axis). As the CNT yarn was further tensioned, the orientation angles of the CNTs decreased significantly, i.e., a bunch of CNTs rotated such that they became more aligned with the tensile axis (Supplementary Figure 3). The average changes of the orientation angles were calculated to quantitatively describe the orientation changes. At small strain (from 0 to 0.009), individual CNTs rotated 2.4° away from the tensile direction. When the strain increased from 0.009 to 0.038 and from 0.038 to 0.063, the orientation angle of the CNTs changed 7.2 and 3.1° toward the tensile direction, respectively. These large angle changes near the yarn surface were not expected at low strains because the orientation angles of individual fibers within a staple yarn should decrease gradually from the yarn surface to the core upon applied tension, according to classical theories of yarn mechanics, e.g., the idealized helical model^14^. Accordingly, other mechanisms (e.g., a rotation of CNT bundles) seem to have been involved during the tensile testing of the CNT yarn, which was confirmed using Raman spectroscopy as described below.

The deformation behavior of CNTs in the CNT yarn was again investigated with CNT yarns having a diameter of about 1 μm. Due to the penetration depth of the Raman laser (about 460 nm), it was expected that such a thin CNT yarn could reveal the deformation behavior of individual CNTs residing on the core part of the yarn. Supplementary Figure 4 shows the orientation angles of individual CNTs of the CNT yarn at different strain levels. The orientation angle of the CNTs in the whole CNT yarn increased at low strain levels, i.e., CNTs were oriented away from the yarn axis (Supplementary Figure 4). As this change was also observed in Supplementary Figure 3, it was attributed to the orientation changes of CNTs near the yarn surface due to tension-induced twist. The CNTs rotated toward the tensile direction as the CNT yarn was axially strained further from 0.008 to 0.033. However, there were few changes in the orientation distribution when the strain was still further increased to 0.046. Therefore, the CNTs near the core of the CNT yarn were not easy to move or rotate, probably due to the lateral pressure, so that the orientation angle change of the CNTs was small.

**Figure 11:**
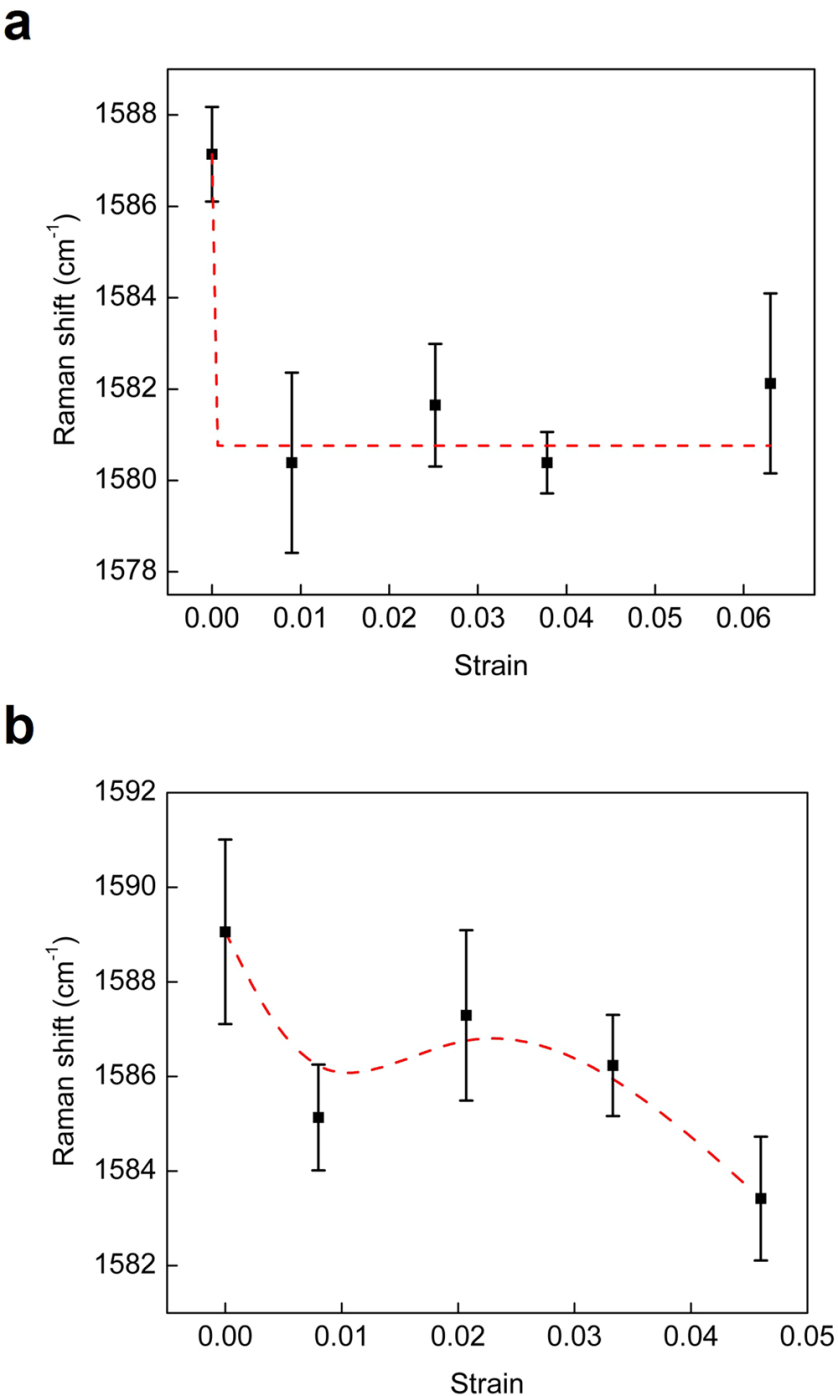
Shift of the G band in the Raman spectra of the CNT yarns during tensile testing as a function of strain. Diameter: (**a**) 10 μm and (**b**) 1 μm.

Raman spectroscopy can characterize the deformation of CNTs under an external force because the shift of the Raman bands depends on the external force, i.e., upward or downward under uniaxial compression or tension, respectively^26^. In this study, the deformation of individual CNTs, in particular those located near the surface of the CNT yarn during the tensile test, was investigated using the shift of the G band (1500–1605 cm^−1^). Figure 11a shows that the shift was large at a low strain (0.009), which implied that the CNTs were actually deformed during the test. As the strain of the CNT yarn increased to 0.038 and further to 0.063, the shift was, however, negligible, which suggested that the CNTs were not stretched at those higher strain levels. The negligible peak shifts at these strains and the orientation change of the CNTs (Supplementary Figure 3) strongly suggest that individual CNTs within the CNT yarn rotated without being stretched after a certain strain of the CNT yarn. The peak shift was also investigated for the thin CNT yarn case. The shift was not as large as with the thick CNT yarn (Fig. 11b). However, the shift continued with increasing strain. This suggested that strain caused stretching of the CNTs in the core of the yarn. It would appear that the CNTs in the center region could sustain the tension continuously due to lateral pressure from the surrounding CNTs.

## Discussion

The deformation behavior of CNT yarns was investigated using *in situ* polarized Raman spectroscopy. Changes in the orientation angles of individual CNTs and the Raman band shift were examined during untwisting and retwisting operations. For the one-ended tethered CNT yarns, a permanent deformation occurred due to snarl formation during untwisting; this resulted in irreversible rotational behavior. For the two-ended tethered yarns, high torques were relieved by untwisting without snarl formation, bringing about reversible rotational behavior. Tension-induced twist could explain the reversible and repeatable rotational behavior of the two-ended tethered CNT yarn. *In situ* tensile testing of CNT yarns within the Raman spectrometer were carried out. These experiments demonstrated that at small strain, the orientation angle of CNTs in the CNT yarn increased, i.e., tension-induced twist was generated, due to the contribution of tensioned CNTs to the yarn torque. As the CNT yarn was further extended, the orientation angle of the CNTs in the yarn decreased due to the lateral pressure and decreased torque. The CNTs on the CNT yarn surface were stretched at small strain and were not further stretched as the deformation proceeded, while the CNTs in the yarn core were continuously stretched. This behavior was explained by the core–sheath structure of twist-spun CNT yarns.

Through this study, we revealed the tension-induced twisting mechanism that can be found only in CNT yarns and that can explain the torsional behavior of CNT yarns regardless of actuation methods. The tension-induced twisting is an unusual phenomenon that cannot be explained by the classical dynamics at large scale because twists are released when conventional yarns are tensioned. It is difficult to realize the twisting motion of material by the linear force due to the unavailability of degree of freedom^27^. Utilizing tension-induced twisting mechanism, twisted CNT nano-fibers in relatively simple structure can be applied to a variety of future sophisticated mechanical systems (requiring twisting motion) including chiral mechanical metamaterials^27–29^ without artificially complex design.

## Methods

### Preparation of CNT yarns

Twist-spun CNT yarns were manufactured using a dry spinning process, which consisted of drawing and twisting a CNT forest. The internal structure of twist-spun CNT yarns varies with radial position because individual CNTs are highly packed in the yarn core. Considering the packing tendency and the penetration depth of the Raman laser, CNT yarns with different diameters were manufactured to observe the internal structure of CNT yarns from their cores to their surfaces when they were actuated to rotate.

CNT forests were provided by the NanoTech Institute of the University of Texas at Dallas. CNTs were grown on an iron-coated silicon substrate by chemical vapor deposition (CVD) at 680 °C. Acetylene (5%) and helium gases were used as the carbon feedstock and the carrier gas, respectively. Their total feed rate was 580 sccm for 10 min. CNT yarns were manufactured from these CNT forests. First, CNT webs were drawn from the CNT forest, maintaining a width of *ca*. 5 mm, and twisting with a rotational speed ranging from 1000 to 4000 rpm. The drawing speed was set at 3 cm min^−1^. Three kinds of CNT yarns (diameters: *ca*. 10, 4 and 1 μm) were manufactured by controlling the rotational speed, e.g., 1000, 2000 and 4000 rpm, respectively.

### *In situ* Raman spectroscopy and penetration depth

Devices were designed to twist/untwist and extend CNT yarns within a predetermined amount (Fig. 1). An *in situ* Raman spectroscopy system was set up by installing those devices in a Raman spectrometer (JASCO NRS-3100). CNT yarns were twisted/untwisted or extended, during which polarized Raman spectra were obtained by excitation at 532 nm. A microscope objective (100×) was used to focus the laser and collect the Raman scattering by the sample. The focused laser was 2 μm and 2.6 mW in size and power, respectively. The Raman data were collected over the range of 1000–2000 cm^−1^ at a resolution of 1.87 cm^−1^.

The intensity of the laser irradiation decreases as it penetrates CNT yarn. This scattering phenomenon can be explained by the Beer–Lambert law, *I* = *I*_0_*e*^*−αz*^, where *I*_0_ is the intensity of the incident light, *α* is the absorption coefficient, and *z* is the travel depth of the laser. The penetration depth of the laser is defined as *z* = *1/α* where the intensity of the laser decays to *1/e*-times its intensity at the surface. The penetration depth of the Raman laser into a CNT yarn was determined to identify the measurement area. Because CNT yarns were manufactured by twisting CNT sheets drawn from a CNT forest, the penetration depth of the Raman laser into the CNT sheets was determined and used as the penetration depth of the Raman laser into a CNT yarn. In detail, changes in the Raman intensity of the silicon peak (originating from the substrate of the CNT sheets) were measured as a function of CNT sheet thickness. The thickness of a CNT sheet was measured using atomic force microscopy (AFM; Park System XE100) operating in the non-contact mode. The scan size and rate of the AFM were 10 μm × 10 μm and 1 Hz, respectively. The density of produced CNT sheets was 0.69 g/cm^3^, while that of CNT yarns was in the range from 0.39 to 0.68 g/cm^3^. The porosities (*φ*) of CNT sheets and yarns were calculated from *φ* = 1−*ρ*_sheet or yarn_/*ρ*_CNT_, where *ρ* is the density of CNT sheets or yarns and *ρ*_CNT_ is the density of CNT (1.76 g/cm^3^)^30^. The calculated porosity value was 0.61 and 0.61 to 0.78 for CNT sheets and yarns, respectively. The incident intensity (*I*_0_) of the silicon wafer was initially measured and the penetration depth was calculated from the intensity of the Raman laser within the CNT sheet (*I*) and the thickness of the CNT sheet (*z*) according to the Beer–Lambert law. The penetration depth of the Raman laser used in this study into the CNT yarns was 465 nm (standard deviation: 194 nm).

### *In situ* torsion and tensile testing of CNT yarns

Twist-spun CNT yarns have a core–sheath structure^31^ in which the packing density of CNTs in the sheath is much lower than in the core. Therefore, CNTs on the sheath affect the torsional behavior of a CNT yarn more than CNTs in the core. Considering the penetration depth of the Raman laser and the sheath part of the CNT yarns (whose thickness is commonly from a quarter to a half of the diameter of the core part)^11^, CNT yarns with a diameter of 4 μm were used to investigate the torsional behavior of CNT yarns. Two boundary conditions were applied to CNT yarns as shown in Fig. 3. The one-ended tethered yarns are known to rotate much higher than the two-ended tethered yarns; however, their rotational behavior is irreversible^7^. CNT yarns (length: 5 cm) were first untwisted (−80 turns cm^−1^) and then retwisted (+80 turns cm^−1^), during which the Raman spectra were obtained to investigate the orientation change of individual CNTs in the CNT yarn. At least 20 samples were tested for each torsion and tensile condition to obtain consistent Raman spectra.

The orientation change of individual CNTs in the CNT yarn when extended was also measured. Two kinds of CNT yarns were used to investigate the internal change of individual CNTs throughout CNT yarns. Due to the penetration depth of the Raman laser, a thick CNT yarn (diameter: 10 μm) was used to investigate the structural changes of the CNT yarns near their surface, while thin CNT yarns (diameter: 1 μm) were used to investigate the internal change in the core of the CNT yarn.

## Electronic supplementary material

Supporting Information
