# Supplementary material for: Tension-induced twist of twist-spun carbon nanotube yarns and its effect on their torsional behavior
Source: Sci Rep. 2018 Apr 18;8:6146. doi: 10.1038/s41598-018-24458-0 (PMC5906567; doi:10.1038/s41598-018-24458-0)
Supplement: Supplementary file 1 — Supporting Information [file 41598_2018_24458_MOESM1_ESM.pdf]

Supporting Information to

Tension-induced twist of twist-spun carbon nanotube yarns and its effect on their torsional behavior

Seung-Yeol Jeon, Dongil Kwon, and Woong-Ryeol Yu[1,*]

Department of Materials Science and Engineering and Research Institute of Advanced Materials (RIAM), Seoul National University, Seoul, KOREA

Orientation and deformation of individual CNTs

The Raman spectra obtained from *in situ* torsion and tensile testing of CNT yarns were analyzed to characterize the orientation and deformation of individual CNTs in the CNT yarn. Two parameters, orientation density function and peak shift, were determined from Raman spectra, brief explanations of which are given below.

The orientation of individual CNTs can be described by the Euler angle (θ, φ, ε), which represents the spatial orientation of an object based on a reference frame (here, CNT yarn) using compounded rotations (Supplementary figure 1).[1] Angles θ, ε, and φ are the polar angle between a CNT and the CNT yarn axis, the azimuthal angle between the *x*-axis of a CNT and the intersection of the xy- and XY-planes (N-axis), and the azimuthal angle between the X- and N-axes, respectively. The orientation distribution function (ODF), $f(\theta,\varphi,\varepsilon)$, is

[1]Corresponding author. Email: woongryu@snu.ac.kr

defined such that $f(\theta,\varphi,\varepsilon)\sin\theta\, d\theta\, d\varphi\, d\varepsilon$ represents the probability of finding an individual CNT in the CNT yarn between (θ, φ, ε) and (θ +dθ, φ +d φ, ε +dε).[2]

By considering the axisymmetric nature of the CNT yarns, it can be assumed that the ODF is independent of the azimuthal angle (φ). Furthermore, CNTs can be assumed to possess cylindrical symmetry, so the ODF is also independent of the angle ε. Therefore, the ODF can be assumed to be a function of only θ, which is the angle between the CNT axis and the CNT yarn axis.[2] Finally, the ODF can be expressed by the maximum entropy formalism as follows:[3,4]

$$f(\theta) = A\exp[-(\lambda_2 P_2(\cos\theta) + \lambda_4 P_4(\cos\theta))] \quad (1)$$

where $P_i(\cos\theta)$ is the Legendre polynomial of degree *i*. Coefficients A, λ_2, and λ_4 are determined to satisfy the characteristics of the ODF, as follows:

$$\begin{aligned}
&\int_0^{2\pi}\int_0^{2\pi}\int_0^{\pi} f(\theta)\sin\theta d\theta d\varphi d\varepsilon = 1\\
&\int_0^{2\pi}\int_0^{2\pi}\int_0^{\pi} P_2(\cos\theta) f(\theta)\sin\theta d\theta d\varphi d\varepsilon = < P_2(\cos\theta) >\\
&\int_0^{2\pi}\int_0^{2\pi}\int_0^{\pi} P_4(\cos\theta) f(\theta)\sin\theta d\theta d\varphi d\varepsilon = < P_4(\cos\theta) >
\end{aligned} \quad (2)$$

where $< P_i(\cos\theta) >$ is the average value of the Legendre polynomial of degree i and determined by the polarized Raman scattering intensity of CNT yarns by solving the following equations: [2]

$$\frac{I^{VV}{}_{CNTyarn}(\Phi=0)}{I^{VH}{}_{CNTyarn}(\Phi=0)}=\frac{I^{VV}{}_{CNTyarn}(\Phi=0)}{I^{VH}{}_{CNTyarn}(\Phi=90)}=-\frac{24<P_4(\cos\theta)>+60<P_2(\cos\theta)>+21}{12<P_4(\cos\theta)>-5<P_2(\cos\theta)>-7}$$
$$\frac{I^{VV}{}_{CNTyarn}(\Phi=90)}{I^{VH}{}_{CNTyarn}(\Phi=0)}=\frac{I^{VV}{}_{CNTyarn}(\Phi=90)}{I^{VH}{}_{CNTyarn}(\Phi=90)}=\frac{-9<P_4(\cos\theta)>+30<P_2(\cos\theta)>-21}{12<P_4(\cos\theta)>-5<P_2(\cos\theta)>-7} \quad (3)$$

Here, I_{VV} and I_{VH} are the intensities obtained from a polarized Raman spectrum in which the polarization direction of the polarizer and analyzer are parallel and perpendicular with respect to each other, respectively. Note that Φ in Eq. (3) is the angle between the CNT yarn axis and the polarizer direction.

The shift of the Raman bands greatly depends on the deformation modes (compressive and tensile modes) of the CNTs,[5] i.e., Raman bands tend to shift upward or downward during uniaxial compression or tension, respectively. The deformation of individual CNTs in the CNT yarns during tension of the yarns was investigated using the shift of the Raman peaks.

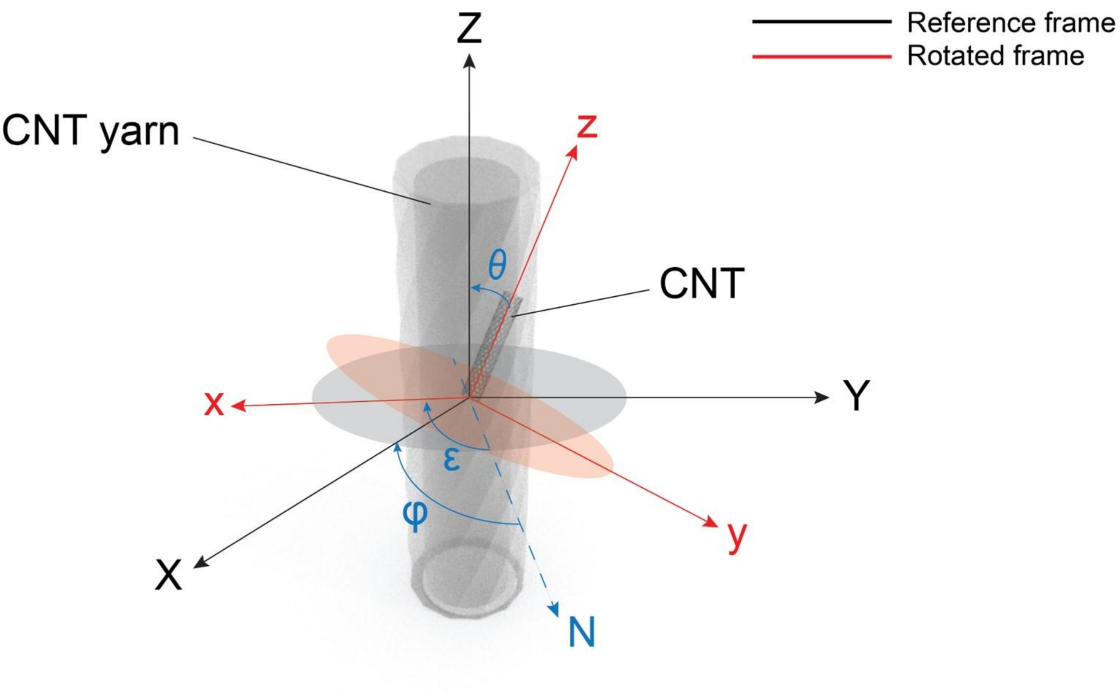

Supplementary figure 1. Euler angles representing the orientation of individual CNTs in the CNT yarn.

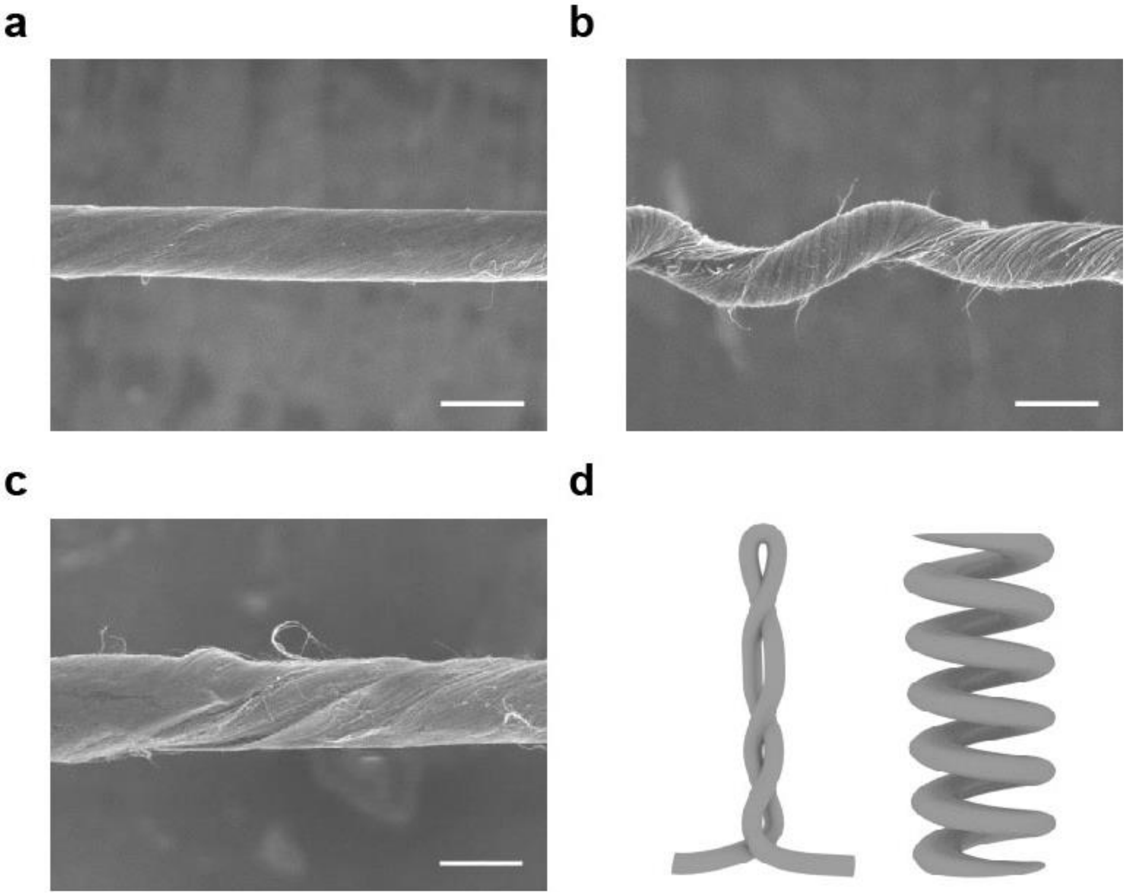

Supplementary figure 2. Morphological changes of a CNT yarn around the fixed region of the one-ended tethered CNT yarn (scale bar represents 10 μm): (a) initial state, (b) after untwisting (−80 turns cm^{-1}), (c) after retwisting (+80 turns cm^{-1}) and (d) a schematic diagram of yarn snarls.

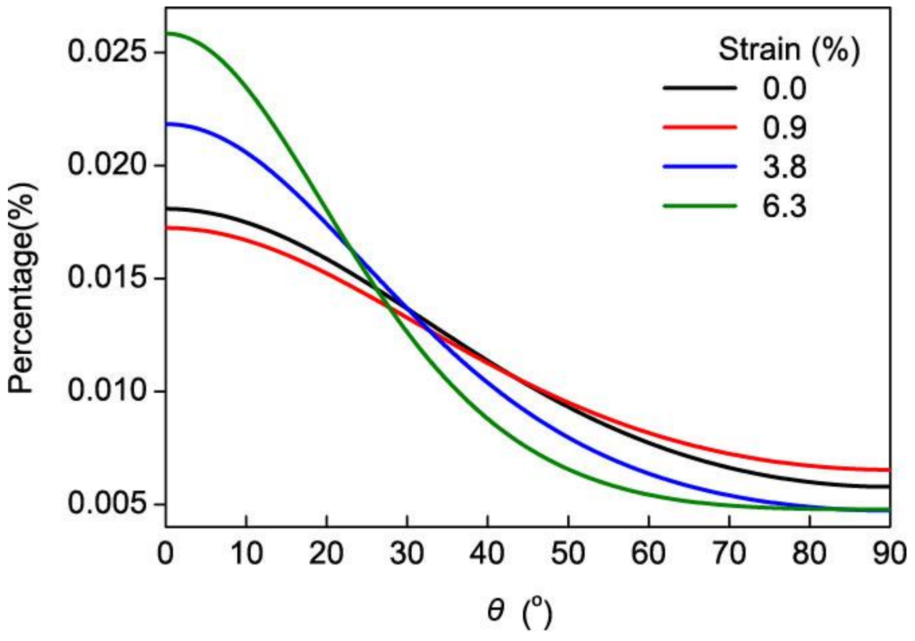

Supplementary figure 3. Distribution of the orientation angle (θ) as a function of the imposed strain of individual CNTs in the CNT yarn having a diameter of 10 μm.

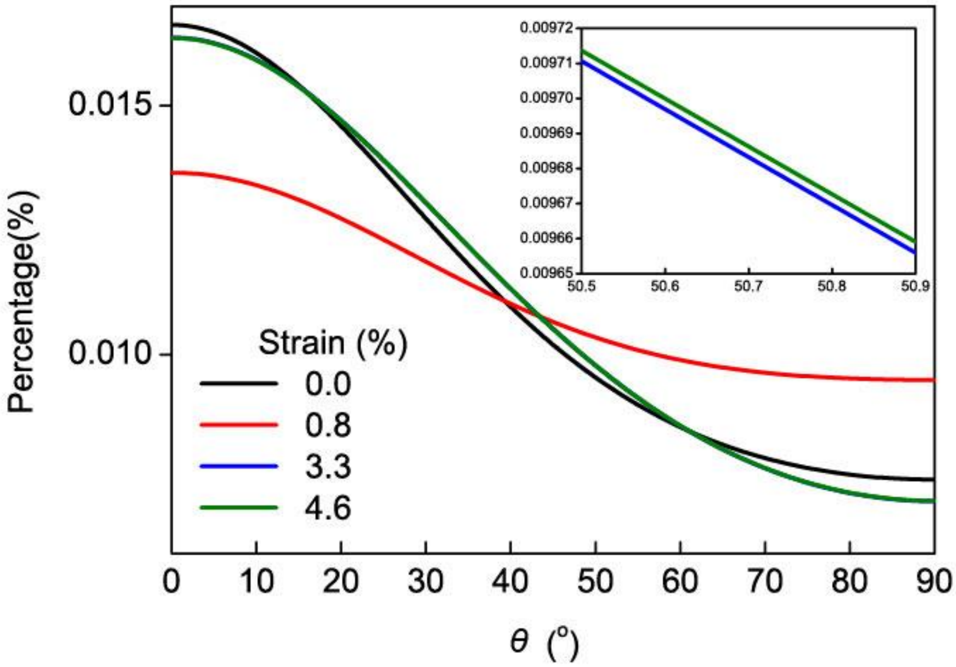

.

Supplementary figure 4. Distribution of the CNT orientation angle of the CNT yarns (diameter: *ca.* 1 μm) as a function of strain level.

References

1 Foroughi, J. *et al*. Torsional Carbon Nanotube Artificial Muscles. *Science* **334**, 494-497 (2011).

2 Liu, T. & Kumar, S. Quantitative characterization of SWNT orientation by polarized Raman spectroscopy. *Chemical Physics Letters* **378**, 257-262 (2003).

3 van Gurp, M. The use of rotation matrices in the mathematical description of molecular orientations in polymers. *Colloid & Polymer Science* **273**, 607-625 (1995).

4 van Gurp, M. Letter to the Editor: On the use of spherical tensors and the maximum entropy method to obtain closure for anisotropic liquids. *Journal of Rheology (1978-present)* **42**, 1269-1271 (1998).

5 Gouadec, G. & Colomban, P. Raman Spectroscopy of nanomaterials: How spectra relate to disorder, particle size and mechanical properties. *Progress in Crystal Growth and Characterization of Materials* **53**, 1-56 (2007).
